# Study of optimal vaccination strategies for early COVID-19 pandemic using an age-structured mathematical model: A case study of the USA

**DOI:** 10.3934/mbe.2023481

**Published:** 2023-04-19

**Authors:** Giulia Luebben, Gilberto González-Parra, Bishop Cervantes

**Affiliations:** Department of Mathematics, New Mexico Tech, New Mexico, 87801, USA.

**Keywords:** Mathematical model simulation, COVID-19, SARS-CoV-2 virus, optimal vaccination, age structure, comorbidity, vaccination hesitancy

## Abstract

In this paper we study different vaccination strategies that could have been implemented for the early COVID-19 pandemic. We use a demographic epidemiological mathematical model based on differential equations in order to investigate the efficacy of a variety of vaccination strategies under limited vaccine supply. We use the number of deaths as the metric to measure the efficacy of each of these strategies. Finding the optimal strategy for the vaccination programs is a complex problem due to the large number of variables that affect the outcomes. The constructed mathematical model takes into account demographic risk factors such as age, comorbidity status and social contacts of the population. We perform simulations to assess the performance of more than three million vaccination strategies which vary depending on the vaccine priority of each group. This study focuses on the scenario corresponding to the early vaccination period in the USA, but can be extended to other countries. The results of this study show the importance of designing an optimal vaccination strategy in order to save human lives. The problem is extremely complex due to the large amount of factors, high dimensionality and nonlinearities. We found that for low/moderate transmission rates the optimal strategy prioritizes high transmission groups, but for high transmission rates, the optimal strategy focuses on groups with high CFRs. The results provide valuable information for the design of optimal vaccination programs. Moreover, the results help to design scientific vaccination guidelines for future pandemics.

## 1. Introduction

At the end of 2020, the U.S. Food and Drug Administration issued an Emergency Use Authorization for COVID-19 vaccines and several countries including the U.S. began a mass vaccination campaign [1]. Vaccination reduces the likelihood to die from Covid disease and therefore it is very important for public health worldwide [2–5]. With an unlimited vaccine supply and resources an ideal situation where everyone can be vaccinated can occur. However, at the end of 2020 and beginning of 2021 there was a highly restricted supply of vaccines against the SARS-CoV-2 virus and therefore vaccination prioritization was needed [6–10]. The issue of vaccine accessibility has been raised by a number of authors [11–15]. Thus, due to the limited availability of vaccines during the early COVID-19 pandemic it is necessary to develop scientific studies in order to find optimal allocation of vaccines [8, 12, 14, 16]. Studies have specifically taken into account the influences of space and time [7, 9, 13]. It has been found that many studies related to the optimization of allocation of vaccines prioritize health care workers and older adults [12]. It has been mentioned that forecasting the COVID-19 pandemic is complex due to many interactions that affect the dynamics [17] For instance, in [18] the author made a comparative analysis and found seasonal behavior in the total environment of COVID-19. Therefore, due to the uncertainty of many factors related to the dynamics of the COVID-19 pandemic it is difficult to have certainty about the optimal allocation of vaccines. An optimal vaccination program can drastically reduce the number of deaths, infected cases and years of life lost. Thus, the development of studies that address or design optimal vaccination programs is of paramount importance for public health worldwide. However, in [18] the results suggest that the increasing share of people vaccinated against COVID-19 seems to be a necessary but not sufficient health policy to reduce the mortality of COVID-19.

Scientific reviews of some previous models that investigated the optimal allocation of vaccines against SARS-CoV-2 have been presented in [8, 12]. Other aspects such as association of doses of vaccines and the General Index of Governance have been investigated [19]. As is expected from modeling studies, there are some limitations on each model or study due to the complexity of the real situation of the COVID-19 pandemic as well as human behavior. However, these studies provide additional insight and a variety of points of view related to the optimal allocation of strategies. Moreover, due to the assumptions related to each modeling study some results or conclusions are more suitable to some countries than others. It is important to remark that before the COVID-19 pandemic similar studies related to the optimization of vaccination programs have been done for influenza [20]. Interestingly, it was concluded that children should be prioritized due to their critical role in the transmission of influenza [3, 21]. In the case of the COVID-19 pandemic this approach was not feasible since at the beginning of the pandemic the vaccines were not approved for children. Another interesting result that was found for influenza vaccination is that direct protection (efficacy against the disease) is superior when reproduction numbers are high but indirect protection (transmission of the virus) is superior when transmission is low [3, 20]. Despite the current COVID-19 pandemic there are studies based on mathematical models related to vaccination campaigns for other diseases [22–25].

Previously, mathematical models have been developed to study the optimal allocation of vaccines which is very important to save human lives [26–31]. Mathematical models are useful due to a variety of reasons. For instance, many different simulations can be performed, allowing the investigation of various factors under a variety of scenarios where uncertainty plays an important role. Some studies have used simple SIR or SEIR models without age-group structure which is a crucial factor for optimal vaccination [32–35]. In [36] the authors formulated an optimal control problem to design vaccination schedules taking into account the number of disability-adjusted years of life lost. They found that a constant vaccination policy is not as good as a policy that assigns a large number of vaccines in the first 20 days. This result agrees in certain aspects with the results presented in [37]. In another study the authors used an age-structured, expanded SEIR model with social contact matrices to evaluate age-specific vaccine allocation strategies [38]. The authors varied vaccine characteristics in their simulations in order to take into account COVID-19 related uncertainty [38]. In [13] the authors proposed a spatial priority-based vaccine strategy for Bangladesh due to the importance of spatial transmissibility. Another work that studied vaccination with spatial effects is presented in [39]. Another interesting study is presented in [9], where the authors used a deterministic mathematical model with limited supply and mass vaccination. They compared the effectiveness of vaccinating health workers, young people and older adults. They found that under some conditions age-based strategies make minimal impact on the epidemic, but vaccinating older people prevents more deaths [9]. This result agrees with that presented in [40] despite the authors using a different approach. We would like to mention the work presented in [3]. In this work the authors evaluated different vaccination strategies using an age-stratified SEIR model. Their model includes information from an age dependent contact matrix and considers a transmission-blocking vaccine. In [31] the authors found that an increase in the proportion of vaccines giving priority to a younger group always had a favorable effect, and prioritizing vaccine allocation among the 60+ age group with 60% of the total amount of vaccine consistently resulted in the greatest reduction in deaths. An interesting work is presented in [41] where spatial prioritization was studied using an agent based model. The authors found that spatial effects are important for allocation of vaccines. All these previous results highlight the importance of COVID-19 vaccine allocation policies under different priority strategies.

Some additional studies that focused on vaccination strategies taking into account different number of doses have been done [42–45]. Delivering a single dose to numerous recipients has been beneficial, according to some investigations [46–48]. For instance in [10] the authors used a SEIR-type model that incorporates COVID-19 asymptomatic and symptomatic infections to evaluate vaccination strategies in terms of infections, hospitalizations, and mortality. One main result of this study was that stretching the between-dose lead time flattens the infection curve and reduces both hospitalizations and mortality compared with the strategy of releasing second doses. In [49] the authors found an interesting result. When minimizing deaths, if the vaccine efficacy is high it is better to allocate vaccines to younger age groups first for high vaccination coverage. This result agrees with those presented in [50] despite the fact that the mathematical models are different. In [51] a nonlinear model was developed to find the optimal scheduling of first and second doses. Their results suggest that the optimal vaccination program depends on the nontrivial scheduling of first and second doses, the efficacy of the first dose to provide partial immunity, waning effects, and the status of the epidemic process.

As we have previously mentioned under limited vaccine supply it is extremely important to design optimal allocation of vaccines. The Centers for Disease Control and Prevention (CDC) designed a vaccination program that allocated vaccines based mainly on work status, age, living conditions and comorbidities [16]. Previous work focused on age as the important factor to decide vaccination strategies. However, other studies have used different factors such as occupation, comorbidities, and social behavior [52]. In [16] the authors developed a model that considered together several characteristics related to the specific CDC recommendations such as age, occupation, comorbidity status, and living condition. In addition, they considered specific vaccine availability in the USA. This aspect differentiates this study from many others where the vaccination pace is generally a constant proportion of the population [32, 34, 35]. The authors analyzed in a clever way all possible vaccine allocation strategies, instead of a few strategies. Obviously this generates a computational issue due to the large number of strategies that need to be tested. The authors found that the CDC recommendation was not the optimal allocation strategy under the assumptions of the mathematical model.

Deciding what should be considered the optimal vaccination program in terms of outcomes is debatable since the goal might be to minimize deaths, infected people, or other variables. In this article, we construct a mathematical model based on nonlinear ordinary differential equations, where each variable represents a different subpopulation with regard to COVID disease progression, vaccination status, comorbidities and age group. The constructed model includes symptomatic and asymptomatic individuals [53–57]. Asymptomatic people are important contributors to the dynamics of the COVID-19 pandemic [58–62]. The impact of asymptomatics on the COVID-19 pandemic has been examined in earlier papers [63–67]. Our model also includes people who are hesitant to be vaccinated. In [68], the authors found results that suggest that COVID-19 vaccine hesitancy may be high among Black immigrants in the U.S. In addition, they found that lower educational attainment, being female, and employment in a health care setting were associated with vaccine refusal and delay.

In this article, one aim is to study vaccination strategies under different scenarios by using a mathematical approach. Another aim is to show the importance of designing an optimal vaccination campaign in order to save human lives. We designed a mathematical model of COVID-19 transmission that considers age, comorbidity status, vaccination status, hesitancy to be vaccinated and disease status since these affect the number of deaths during the COVID-19 pandemic. In this study we take into account a social contact matrix. This differs from many previous studies. The model structure is designed to resemble the real situation in the early stage of the vaccination program against SARS-CoV-2 in the USA. The mathematical approach allows us to study infinitely many scenarios with different transmission rates of SARS-CoV-2. Thus, with this study we are able to provide additional scientific insight for the importance of vaccination programs and optimal vaccination strategies. The mathematical approach presented in this paper can be also used for future pandemics depending on their particular features. For instance, future pandemics might have different scenarios due to factors such as governance, health expenditures and pandemic prediction [69].

It is important to mention that the aim of this study is not to precisely forecast the dynamics of the COVID-19 pandemic. Rather, our goal is to understand and investigate the effects of implementing a variety of vaccination strategies on crucial public health outcomes, such as the number of deaths. We use the number of deaths as a metric to compare vaccination strategies, but other metrics can be used or combined. We tested all priority-order vaccination strategies than can be implemented with the population structure that the our mathematical model has. Considering all combinations we can assure that we obtain the optimal vaccination strategy under the population structure conditions imposed by the mathematical model and using priority-order vaccination strategies. In some ways an order priority vaccination strategy can be seen as one particular path to vaccinate target groups. In general, a particular strategy would generate different infected cases and numbers of deaths. In this study we try to consider the main factors that affect the relevant outcomes. However, the real world includes many other factors to consider. Including all of them in a mathematical model approach research is complex and it might give a more complicated picture to understand the effects of all those factors on a vaccination program [17, 70–73]. In this study we consider only scenarios where the vaccination pace is that used in the USA during 2021 at the beginning of the vaccination program. This greatly differs from other work in which proportional vaccination rates have been used [7, 9, 26, 29, 31]. However, there is an important study where they used the vaccination pace of the USA used in 2020 [16]. That work used a different population structure with four different age groups and took into account job type and living situation. Thus, they obtained a model based on 340 differential equations which requires 340 initial conditions. They fitted their model using an approximation of the CDC strategy and some assumptions to obtain an estimated transmission rate. Using a specific vaccination pace implies using a mathematical model for short dynamics and classical theoretical stability analysis cannot be done. Thus, from a strictly theoretical mathematical viewpoint this is less interesting, but the model is much more realistic which is a main aim of mathematical approaches to real life situations. It is important to mention that at the beginning of the COVID-19 pandemic there was a huge problem related to vaccine availability and therefore it was of paramount importance to choose optimal vaccination strategies to save lives.

This paper is organized as follows: In Section 2, we present the constructed model and the main assumptions. In Section 3, we present the results in terms of the metric in order to compare the different vaccination programs. In Section 4, we present discussions related to this study and previous work. Finally, in Section 5, we present the main results and conclusions.

## 2. Materials and methods

The constructed mathematical model is based on a nonlinear system of ordinary differential equations with non-homogeneous terms due to the time-varying vaccination pace in the USA during 2021. The model divides the population into mutually exclusive subpopulations. These subpopulations are based on disease status, age group, comorbidity status and vaccination status. The following subpopulations are considered with regard to disease status: susceptible, infected (able to infect others), asymptomatic (able to infect others) and recovered (not infectious). With regard to comorbidity we consider just two statuses: zero comorbidities and one or more comorbidities. Regarding the crucial aspect related to age we consider five age groups: 0–39 years old, 40–59 years old, 60–69 years old, 70–79 years old and 80+ years old. We decided to use the case fatality rate (CFR) to choose the age groups. For instance, we divide the population by age when there is a great variability of the CFR from one age to another. Using age groups by one year difference would create a model with a much larger number of variables and the main results would then be more difficult to interpret. There are only two statuses related to vaccine hesitancy: willing to be vaccinated and vaccine hesitant, although others could be considered, but we are trying to keep the mathematical model relatively simple for many different reasons that will become evident later in this study. The mathematical model allows the movement of people through the aforementioned subpopulations. The model assumes that recovered individuals have permanent immunity against reinfection during the short period of study (180 days) [9]. This is a reasonable assumption since the percentage of breakthrough cases before one year is very low. The model includes the assumption that only susceptibles can take a vaccine dose during the period of study.

The individuals can move from the susceptible subpopulation to vaccinated if they receive a vaccine dose. The model considers the possibility of infection for vaccinated people. However, the chance of infection is greatly reduced by a factor of 1−ε. This has been a standard assumption in other studies [16, 45, 74–76]. There are other possibilities to include the effect of vaccination, for example reduced symptoms. The mathematical model also considers that only symptomatic individuals can die due to Covid disease [77–80].

### 2.1. Mathematical model considering vaccination

The constructed mathematical model is given by the following system of differential equations $\dot{X}(t)=F(X(t))$, where the vector

$$X(t)=\left(S\;h_{ik}(t),S\;w_{ik}(t),S\;v_{ik}(t),I_{ik}(t),Iv_{ik}(t),Ah_{ik}(t),Aw_{ik}(t),Av_{ik}(t),R(t)\right)\in R^{82}$$

and the force of infection is given by

(1) $$\lambda (t)=\frac{1}{N}\sum_{i=1}^{5}\sum_{k=0}^{1}\beta_{ik}\left(Ah_{ik}+I_{ik}+Aw_{ik}+Iv_{ik}+Av_{ik}\right).$$

Then, the mathematical model can be written as

(2) $$\begin{array}{ll} & \dot{S}\;h_{ik}\left(t\right)\;=\;-\lambda \left(t\right)\;S\;h_{ik}\left(t\right),\\ & \dot{S}\;w_{ik}\left(t\right)\;=\;-\lambda \left(t\right)\;S\;w_{ik}-v\left(t\right)\;S\;w_{ik}\left(t\right),\\ & \dot{S\;}v_{ik}\left(t\right)\;=\;-\left(1-\epsilon \right)\lambda \left(t\right)\;S\;v_{ik}\left(t\right)\;+\;v\left(t\right)\;S\;w_{ik}\left(t\right),\\ & {\dot{I}}_{ik}\left(t\right)\;=\;\left(1-a\right)\lambda \left(t\right)\left(S\;h_{ik}\left(t\right)\;+\;Sw_{ik}\left(t\right)\right)-\gamma I_{ik}\left(t\right),\\ & \dot{I}v_{ik}\left(t\right)\;=\;\left(1-\epsilon \right)\left(1-a\right)\lambda \left(t\right)S\;v_{ik}\left(t\right)-\gamma Iv_{ik}\left(t\right),\\ & \dot{A}h_{ik}\left(t\right)\;=\;a\;\lambda \left(t\right)S\;h_{ik}\left(t\right)-\gamma Ah_{ik}\left(t\right),\\ & \dot{A}w_{ik}\left(t\right)\;=\;a\;\lambda \left(t\right)S\;w_{ik}\left(t\right)-\gamma Aw_{ik}\left(t\right),\\ & \dot{A}v_{ik}\left(t\right)\;=\;\left(1-\epsilon \right)a\;\lambda \left(t\right)S\;v_{ik}\left(t\right)-\gamma Av_{ik}\left(t\right),\\ & \dot{R}(t)\;=\;\gamma \left[Ah_{ik}\left(t\right)\;+\;Aw_{ik}\left(t\right)\;+\;Av_{ik}\left(t\right)\;+\;\left(1-\delta_{ik}\right)I_{ik}\left(t\right)\right.\\ & \left.\;+\;\left(1-\delta_{ik}\right)Iv_{ik}(t)\right],\\ & \dot{D}(t)\;=\;\gamma \delta_{ik}\left[I_{ik}(t)\;+\;Iv_{ik}(t)\right], \end{array}$$

where i,k represent the indexes of the ten following groups:

**Table:** 

Age (years)		Comorbidities	
i = 1:	0 – 39	k = 0:	0 Comorbidities
i = 2:	40–59	k = 1:	1+ Comorbidities
i = 3:	60–69		
i = 4:	70–79		
i = 5:	80+		

**Figure 1.**
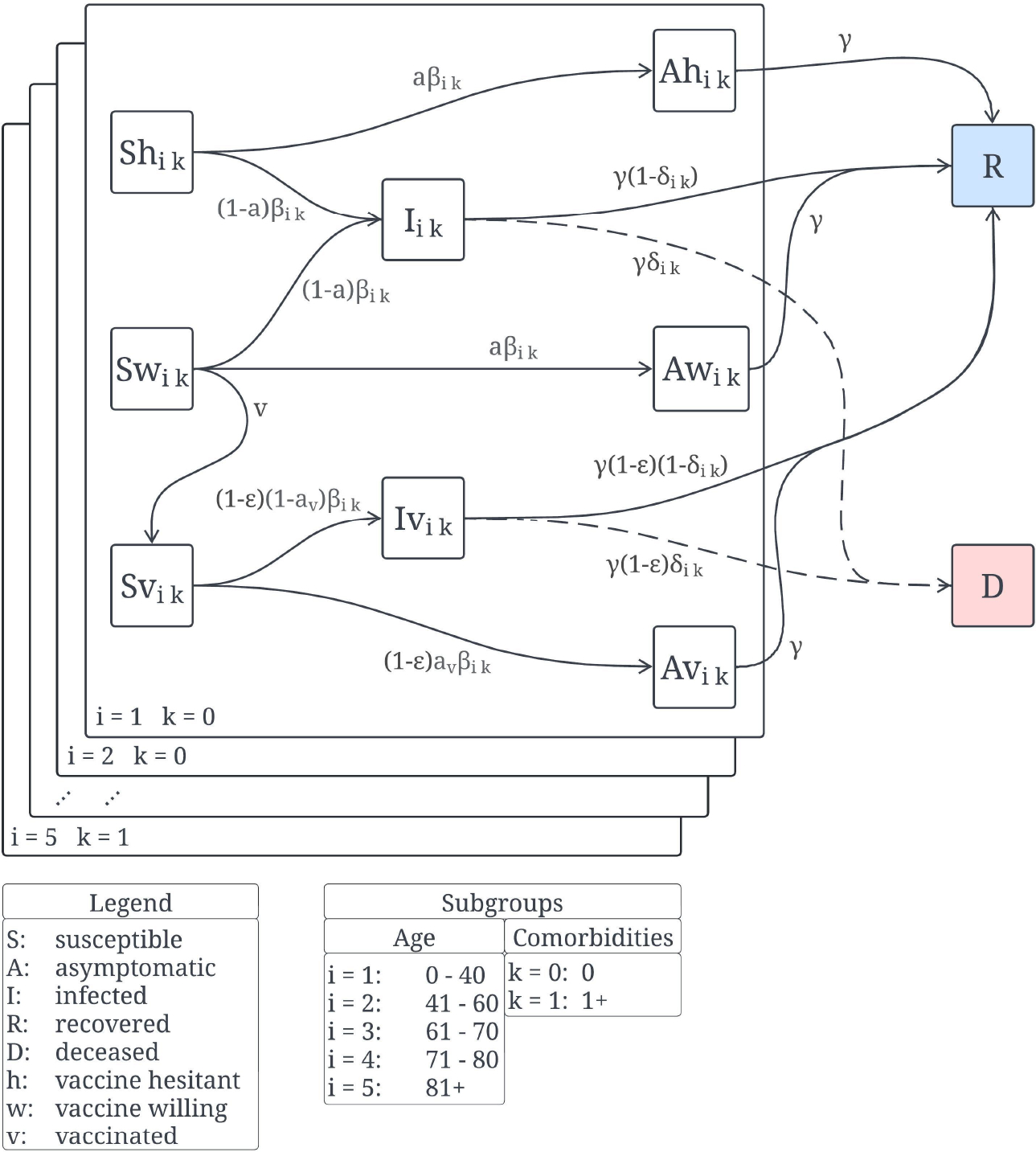
Diagram for the COVID-19 mathematical model (2).

The initial conditions can be written as

(3) $$S\;h_{ik}\left(0\right),\;S\;w_{ik}\left(0\right),\;S\;v_{ik}\left(0\right),\;I_{ik}\left(0\right),\;Iv_{ik}\left(0\right),\;Ah_{ik}\left(0\right),\;Aw_{ik}\left(0\right),\;Av_{ik}\left(0\right),\;R\left(0\right),\;D\left(0\right),$$

where the variable D denotes the number of deaths for the period of study. The number of deaths can be computed by the following ordinary differential equation $\dot{D}(t)=\gamma \delta_{ik}\left[I_{ik}(t)+Iv_{ik}(t)\right]$, represents the number of deaths caused by the disease. The force of infection λ(t) describes the rate at which a susceptible individual becomes infected through interaction with an infected or asymptomatic individual who can transmit SARS-CoV-2. The state variable, $S\;h_{ik}(t)$, represents the susceptible subpopulation hesitant to vaccinate from age group i and comorbidity status k. The state variable, $S\;w_{ik}(t)$, represents the susceptible subpopulation willing to vaccinate from age group i and comorbidity status k. The state variable, $S\;v_{ik}(t)$, represents the susceptible vaccinated subpopulation from age group i and comorbidity status k. In an analogous way we have the state variables $Ah_{ik}(t)$, $Aw_{ik}(t)$ and $Av_{ik}(t)$ which represent the asymptomatic individuals who are hesitant (to be vaccinated), willing (to be vaccinated), and vaccinated respectively. Regarding the variables $I_{ik}(t)$ and $Iv_{ik}(t)$, these represent the non-vaccinated infected subpopulations (willing and hesitant) and vaccinated, respectively. Finally, R(t) represents the subpopulation of recovered people. Figure 1 shows the flows between the different subpopulations depending on the vaccination status, age group, disease and comorbidity status of the people.

### 2.2. Vaccination rate v(t) in the USA for the mathematical model

**Figure 2.**
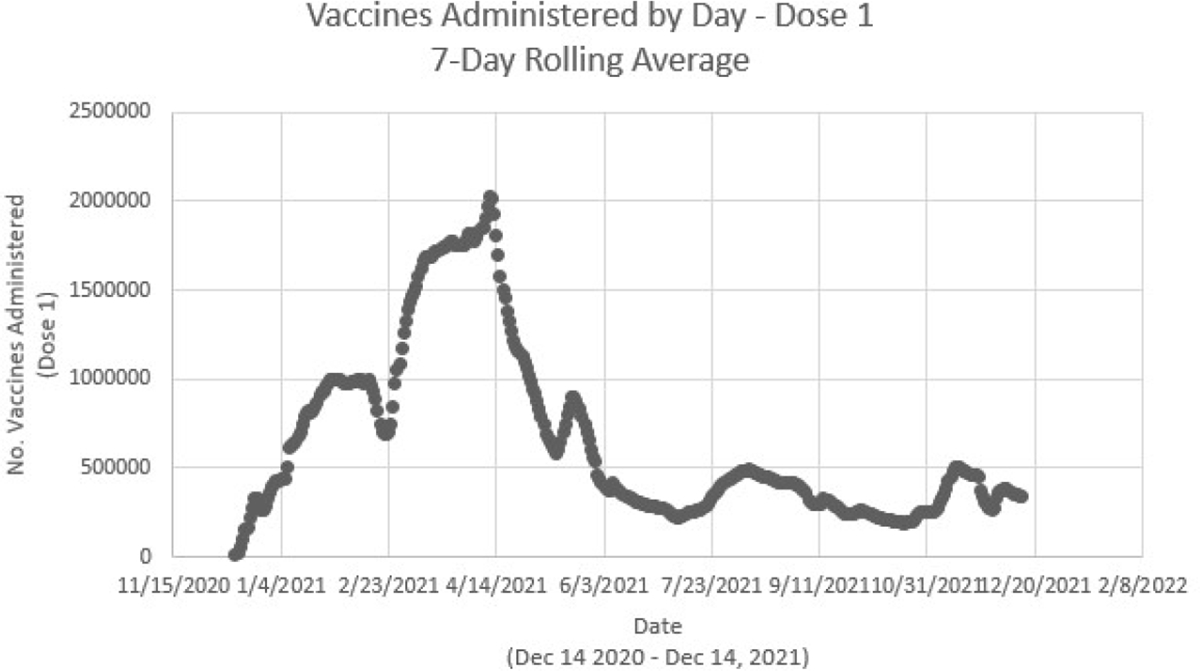
Number of vaccine doses administered per day in the USA from the beginning of the vaccination program until December of 2021 [81].

The constructed mathematical model disregards natural births and deaths, since the study is focused on a short period at the beginning of the vaccination program. The model includes a vaccine efficacy which can be easily modified. The model includes a specific time varying vaccination deployment v(t) for the USA. It is important to remark that in reality a great part of the population will not be vaccinated (hesitant to be vaccinated). Figure 2 shows the number of vaccine doses administered per day in the USA from the beginning of the vaccination program until December of 2021.

### 2.3. Transmission rates and social contact matrix

Regarding the force of infection it is important to mention some crucial details related to the mathematical model (2). The core of the dynamics of the model is the force of infection as is common in epidemiological mathematical models. The force of infection is given by $\lambda (t)=\frac{1}{N}\sum_{i=1}^{5}\;\sum_{k=0}^{1}\;\beta_{ik}\left(Ah_{ik}\;+\right.\left.{\;I}_{ik}\;+\;Aw_{ik}\;+\;Iv_{ik}\;+\;Av_{ik}\right)$. This force of infection in some way measures the likelihood that a susceptible individual gets infected during the COVID-19 pandemic. Notice that the force of infection includes the transmission rate $\beta_{ik}$ which has been assumed to be different for each subpopulation. In reality each person has a different likelihood to get infected, but including this in a model would make the model unmanageable. Approaches to deal with this likelihood uniqueness of each person in epidemics (including COVID) has been approximated using agent based models [82–86]. Agent-based models have been utilized to study the COVID-19 pandemic [87–91]. Agent-based models in particular have been used to examine the vaccination process during the COVID-19 epidemic [41, 46, 92–94].

In this work we use a more classical mathematical approach by means of differential equations where behaviors of different persons are averaged. However, despite the averaged approach each sub-group has its own averaged behavior and its own transmission rate $\beta_{ik}$. This parameter depends on the contact rate (social behavior) and the probability that a contact causes an infection. This probability obviously depends on the infectivity of the particular SARS-CoV-2 variant and the immunity of the person [95–99]. Studies have looked into the consequences of novel SARS-CoV-2 variants [100–102]. In the classical mathematical approach, the probability of infection can be averaged for each subpopulation taking into account the prevalent circulating SARS-CoV-2 variant. However, the contact rate is highly variable and therefore has an intrinsic uncertainty. Many studies have shown this using an estimation of the basic reproduction number ${ℛ}_{0}$ for different regions or countries [103–106].Weather and spatial effects on the basic reproduction number ${ℛ}_{0}$ have been studied [107, 108]. For instance, in [109] the authors found that estimates of the basic reproduction number obtained using Bayesian inference varied from 7.1 for New Jersey to 2.3 for Wyoming. It is important to remark that the basic reproduction number depends on contacts per unit time. Social contact matrices have been used to model the dynamics of different infectious diseases and for the current COVID-19 pandemic [110–115]. In this study we also rely on a social contact matrix in order to have an estimation of the averaged contacts of each subpopulation [116]. For instance, younger people are more reluctant to stay at home or take preventive measures [14]. Therefore, the transmission rate of younger groups is higher in the simulations. Additionally, the transmission rate also varies between specific groups. Since young people are more likely to socialize among their peers than with older adults, the transmission rate between groups of the same age is higher than that of two groups of different ages [116]. Another aspect of the simulations is that older groups and groups with comorbidities have a higher case fatality rate [14]. These last two aspects are based on facts or results of previous studies [117–119].

Another factor that the constructed model considers is that vaccinated people have much lower probability to get infected due to vaccine protection. Furthermore, the model takes into account that, besides the difference in social contacts due to age, there is also a factor related to people who are hesitant to be vaccinated. We assume that these people are also less likely to follow behavior guidelines to avoid COVID infection. Vaccine hesitant individuals were assumed to have a transmission rate of 1.5 times that of their willing and vaccinated counterparts due the correlation between social distancing and increased vaccine willingness [120]. We also assumed that people with one or more comorbidities are more likely to follow behavior guidelines and therefore their transmission rate is reduced. During the post-2020 holiday wave of COVID, individuals without comorbidities had 1.28 times more contacts than individuals with comorbidities so the number of contacts among those with no comorbidities were increased by a factor of 1.28 [121].

Another assumption that we make and which has been used in other studies is the fact that asymptomatic people have different social behavior than symptomatic ones. This model also factors in the differences in social contacts between different comorbidity statuses, as those with high-risk comorbidities are more likely to exercise caution and social distancing [121].

We calculated transmission rates among demographic groups, β, as the product of the contacts between demographic groups and the disease transmissibility. The number of contacts between groups was derived from a social contact matrix with 5 year age groups [116]. The contact matrix was symmetrized and transformed into a 5-by-5 matrix to align with the age groups used in this model. Due to a lack of contact data for individuals over the age of 80, we assumed that their number of contacts with each group is one third of that of the previous age group (70–79 years). We also assumed that there is a 25% increase from the number of contacts between an individual in the 70–79 category and an individual in the 80+ category to two individuals both in the 80+ category. During the post-2020 holiday wave of COVID-19, individuals without comorbidities had 1.28 times more contacts than individuals with comorbidities [121] so the number of contacts among those with no comorbidities was increased by a factor of 1.28. The entire contact matrix was scaled down such that this comorbidity contact factor did not inflate the total number of contacts. We determined a base-transmissibility value, $\beta_{\mathrm{base}}=0.1694$ which was multiplied with the number of contacts for each demographic group in order to determine transmission rates between each group. This value was estimated by using the least-squares method, i.e. this $\beta_{\mathrm{base}}$ parameter minimizes the residuals of the number of cumulative deaths each week provided by the mathematical model (2) and CDC death data [122]. For this least-squares fitting we chose the vaccination strategy that is closest to that of the CDC. For sensitivity analysis purposes we varied in the numerical simulations this value by ±0.05.

### 2.4. Case fatality rates (CFRs)

We use different fatality rates depending on age, comorbidities and vaccination status, which have been used (and estimated) extensively in other studies related to the dynamics of the COVID-19 pandemic [16, 110, 119, 123, 124]. These rates have been estimated in different countries [125–129].

In particular, we calculated the case fatality ratio, δ, for each demographic group [130]. By converting CDC data for the number of deaths per 1,000,000 infections into a proportion, we found the national average CFR to be 0.016. However, infections are more fatal among older individuals and those with comorbidities. The odds ratios (OR) for having a fatal COVID-19 infection have been estimated as 1, 2.53, 7.18, 16.08, and 43.21 for the age groups 0–39 years old, 40–59 years old, 60–69 years old, 70–79 years old and 80+ years old respectively [119].

Individuals with one or more comorbidities have a CFR increased by 1.97 times [117, 118]. This value was calculated by averaging two literature values for the ORs of COVID-19 mortality for individuals with 1, 2, or 3+ comorbidities. It was assumed that among individuals with at least two comorbidities, the proportion of individuals with exactly 2 or 3+ comorbidities was even. CDC data for the prevalence of single and multiple chronic conditions was used to find the weighted average of these ORs in order to determine the value for individuals with one or more comorbidities [131]. We assumed that the cumulative effect of age and number of comorbidities on a demographic’s CFR is the product of the OR for age and the OR for comorbidities.

Next, we calculated the base-CFR, $\delta_{\mathrm{base}}=0.0019$, which is the CFR for the group of individuals aged 0–39 years with no comorbidities (reference group). For all other demographic groups, we found the CFR to be the product of this base-CFR and the aggregate OR for that group’s COVID risk factors. We calculated the base-CFR so that the weighted average matches the US national average CFR of 0.016.

### 2.5. Further parameter values and uncertainty

With regard to other parameters of the model (2) we use parameter values extracted from scientific articles and the CDC’s website. However, there is still uncertainty in the literature. For instance, many previous articles have a high variability for the proportion of asymptomatic infections. The outcome of each vaccination strategy depends on many factors as well as the parameter values. Analyzing vaccination strategies that take into account, in detail, all the uncertain factors is a very complex problem. In this work we perform the analysis focusing on the prioritization of allocation of vaccines to different subpopulations. We studied different scenarios varying the transmission rates to take into account uncertainty in this rate due to the introduction of SARS-CoV-2 variants, the change of social behavior over the period of study and uncertainty in the estimation of the transmission rate. However, for the numerical simulation of each scenario the transmission rates are time-invariant. The vaccination pace is not varied since we use the specific value that was used in the USA using CDC data to determine the exact number of vaccines allocated each day at the beginning of the vaccination program which takes into account the limited supply and further logistic considerations. For the numerical simulations we assumed a constant averaged transmission rate of SARS-CoV-2. This is a mild assumption since the COVID-19 pandemic was already established for more than a year and there was no huge novelty besides the introduction of new variants and the vaccines. These last two factors might have opposite effects on human behavior. The former brings more caution and the second gives more freedom to social behavior. In fact, it has been pointed out that people might change behavior (on average) when a vaccination program is implemented due to a perception of less risk [52]. In addition, for the simulations we assumed that social contacts would not change because many non-pharmaceutical interventions would have been already long implemented before the vaccination program had begun.

### 2.6. Initial conditions for the subpopulations

For the situation of the USA from December of 2020 to June 2021 we considered initial conditions taken from published data, even though not all the initial conditions for each of the subpopulations are available. For initial subpopulations that were not clear, we assumed proportions based on real demographic data and information available in scientific articles [122, 132, 133]. For instance, for the group of individuals older than 80 years with one or more comorbidities we used an initial population of 11,126,210. This was derived from US Census data that indicates the US population above the age of 80 is 12,701,153 [134] and CDC data that indicates 87.6% of these 80+ year old individuals have one or more chronic conditions [131]. We used census data to determine the populations of each age group [134] and CDC data to determine the proportion of individuals in each age group with one or more of the following chronic conditions: arthritis, cancer, chronic obstructive pulmonary disease (COPD), coronary heart disease, current asthma, diabetes, hepatitis, hypertension, stroke, and weak or failing kidneys. [131]. Unvaccinated susceptible asymptomatic groups were subdivided into vaccine hesitant and willing to be vaccinated. Infected groups did not have hesitant and willing categories due to the assumption that a symptomatic individual is more likely to stay at home while experiencing symptoms regardless of their COVID-19 ideology. Those who had not received their first dose of the vaccine by December 14, 2021, one year after the beginning of the vaccine roll-out, according to CDC vaccination data [135], were considered to be vaccine hesitant, as this offered ample time for most people to receive their first vaccination. This approach may overestimate the percent of vaccine hesitant children, as most individuals aged 5 to 11 were not permitted to be vaccinated until November 2021, and children under 5 were not approved for vaccination until after December 14, 2021 [136, 137]. We used CDC case data from the week of December 12, 2020, just as the first vaccines were released, to determine the number of individuals in each group infected with COVID-19 [122]. 30% of these cases were considered to be asymptomatic [130]. We also used CDC data to determine the number of people in each demographic group who had contracted COVID-19 prior to December 12, 2020, putting them in the recovered compartment [122]. In Table 1 we present the specific initial conditions. As we have shown above, the subpopulations have been computed using demographic statistics and taking into account data regarding infected cases and recovered cases [122, 133]. The initial vaccinated population for each age group was taken as zero since the study focuses at the beginning of the vaccination campaign.

## 3. Results

We perform numerical simulations varying the base transmission rate which affects all the transmission rates $\beta_{ik}$ of each susceptible subpopulation. These variations have the objective to assess potential changes due to different transmission rates due to, for instance, the introduction of different SARS-CoV-2 variants and to also account for the fact that the base transmission rate is an estimation of the real one. In addition, we simulated all the different potential strategies that can be implemented based on the constructed mathematical model (2). The total number of potential vaccination strategies that might be implemented using the mathematical model and assuming vaccination by strictly prioritization groups are 3,628,800 which is the total possible number of permutations of the ten demographic subpopulations. Further strategies can be implemented with a model that has more subpopulations, but the computational complexity would be greater. Moreover, if the vaccination strategies also consider the possibility of simultaneous vaccination of different subpopulations under limited vaccines, then the potential strategies are infinitely many, due to the continuity of the proportion values related to the simultaneous vaccination. Thus, we can see that finding the optimal allocation of vaccines is a very complex problem and in this study we provide additional insight about optimal vaccination strategies. We used the number of deaths as the metric to estimate the effectiveness of a given strategy. The optimal vaccination strategy is that which produces the minimum number of deaths from among all the strategies. It is important to point out that the general results of this study are qualitative and are not a description of the past dynamics of COVID-19 in the USA. However, the approach used here can be applied to other countries by changing some parameter values, social contact matrices and initial conditions.

### 3.1. Set up for the numerical simulations

As we mentioned before, we use the number of deaths as the metric to compare the different strategies, even though other metrics, such as infected cases or years of life lost (YLL), could be used. We use the cumulative number of deaths since it is the simplest worst-case COVID-19 outcome. It seems more relevant and years of life lost may be considered controversial [138, 139]. The numerical simulations are performed using the mathematical model (2) and varying the order of the priority groups to be vaccinated. For instance, in one simulation the subpopulation of individuals over the age of 80 with one or more comorbidities may be the first group to be vaccinated. In this case, vaccines will be allocated to this group each day, based on the actual number of first doses of the vaccine administered per day in the U.S., until all individuals in the susceptible willing category have moved to the susceptible vaccinated category, or the infected/asymptomatic compartments. Then vaccines will be administered to the next priority group.

**Figure 3.**
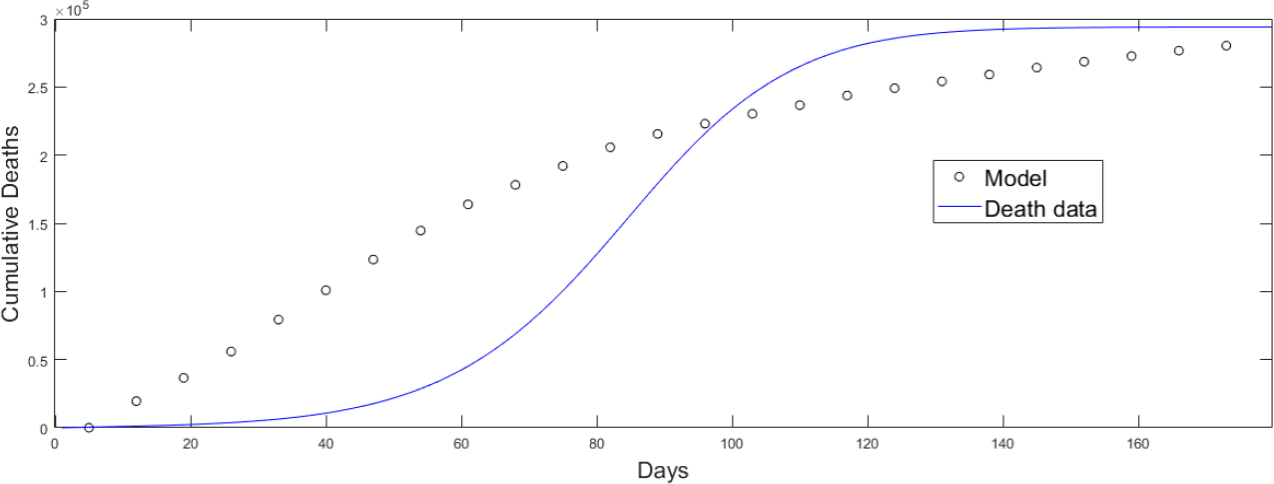
Fitting of the mathematical model (2) to the real data related to deaths in order to obtain a base transmission rate [81].

The estimation of the base transmission rate is based on a fitting process of the mathematical model (2) to real data of the number of deaths. The population structure of the model is different from that used by the CDC to assign the vaccines. Moreover, the CDC used vaccination by phases where several subpopulations were vaccinated in the same phase. Since the CDC vaccination program was the one used in reality, we chose among all the feasible (based on the model (2) vaccination strategies the one closest to the vaccination roll out implemented in the USA in order to fit the model [81]. Due to the large number of parameters these cannot be estimated in a unique way and some assumptions need to be made [16, 50, 140–142]. The fitting process allows us to have an approximate value for the transmission rate and to be able to perform the numerical simulations in a reasonable range. The fitting process can be done in many ways, but we use a standard fitting by minimizing the least square error or sum of squared errors (SSRs). With this process we only estimated the base transmission rate since estimating further parameters with the available data would not provide uniqueness of the solution [50, 142–144]. Fig. 3 shows the fitting of the mathematical model (2) to the real data related to deaths which is more accurate than infected cases. As expected, the fitting is just an approximation that allows us to obtain an estimation of the base transmission rate and to obtain qualitative results. The fitting process uses our mathematical model (2), which has a different population structure than the one CDC used for the inoculation of vaccines and therefore an accurate fit is not expected. Better fits can be obtained at the expense of losing identifiability of the parameters which adds more complexity to the study. All the transmission rates $\beta_{ik}$ are based on the base transmission rate since the transmissibility of the different subpopulations are interrelated. For instance, all the transmission rates depend on the SARS-CoV-2 variants circulating at the time of the implementation of the vaccination program as well as on the social environment over the same time. For example, if the USA government were to remove a non-pharmaceutical intervention that would affect all the different subpopulations and their respective transmissibilities. Considering time-variant parameters would make this study even more complex with a huge number of variations for the vaccination strategies.

In the numerical simulations, for each strategy we computed the total number of deaths under a variety of transmission rates $\beta_{ik}$ for the different subpopulations. The cumulative number of deaths is given by the state variable D(t), which is computed by numerically solving the mathematical model (2). All numerical simulations were carried out in Matlab. The ordinary differential equations were solved using the Euler’s method. The simulations were performed with different computers in order to expedite the computation time since the number of strategies makes the total computation time large.

For all scenarios we fixed the efficacy of the vaccine at 90% in order to focus on the relevant transmission rate of SARS-CoV-2. Table 1 shows the initial conditions for each of the subpopulations [122, 134, 135] and Table 2 the numerical values of the parameters of the mathematical model (2) [130, 145]. One important aspect for the simulations is the CFR for each subpopulation. Table 3 shows the CFRs for each age group depending on the comorbidity status of people. Finally, in Table 4 we present the transmission rates between subpopulations. These numerical values of the parameters were used for all the numerical simulations and testing of strategies in order to have a fair comparison. The only value that was varied is the base transmission rate and as mentioned before this affects all the transmission rates $\beta_{ik}$ as would be expected. The numerical simulations are for 180 days or 6 months. The simulations can be extended for a longer time, but then the mathematical model might need modifications since after six months some conditions may change significantly and certain assumptions made due to the model’s short time span may no longer be valid. For instance, the accuracy of results of longer simulations may be affected by the waning of vaccine protection and natural immunity protection [32]. The transmission rates may have changed due to the introduction of additional SARS-CoV-2 variants with higher transmissibility and therefore the transmission rate would change and/or the model would need to include multiple SARS-CoV-2 variants [146–148]. Some of these factors relating to the emergence of new SARS-CoV-2 variants have been mentioned in a number of studies [149–152].

### 3.2. Numerical simulations for the base transmission rate scenario

**Figure 4.**
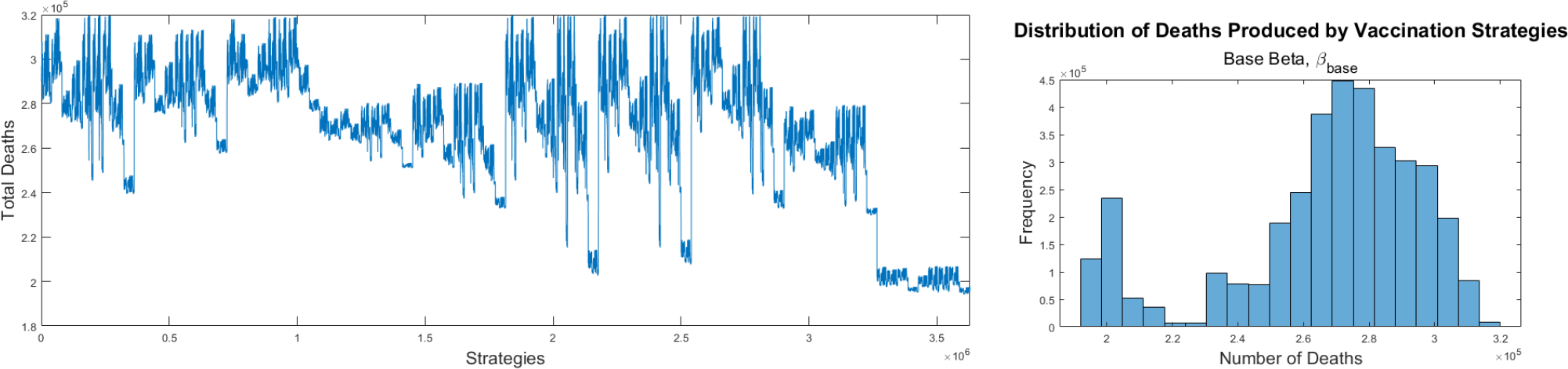
Final cumulative number of deaths for all the different strategies for COVID-19 pandemic vaccination programs using the base transmission rate β≈0.16 (left). Histogram for the different vaccination strategies with regard to the total number of deaths (right).

The first set of numerical simulations that we perform uses the base transmission rate that is obtained from the fitting process to real data of the number of deaths in the USA [122]. All the 3,628,800 potential strategies related to the mathematical model (2) are evaluated using the final cumulative number of deaths after 180 days. The simulations are performed using exactly the same number of vaccines and allocation rates that were used in the USA [81]. This implicitly means that the availability of vaccine against SARS-CoV-2 were limited. This aspect is very relevant to this study and differs with a large number of studies related to optimal vaccine allocation where a proportional vaccination rate is assumed [7, 9, 26, 29, 31]. In addition, the numerical simulations include the fact that the vaccine hesitant population will not be vaccinated as the mathematical model (2) states. Figure 4 shows the final cumulative number of deaths for all the different 3,628,800 potential strategies for COVID-19 pandemic vaccination programs using the base transmission rate β≈0.16. In general, different outcomes are obtained depending on the vaccination strategy. This aspect is crucial from a public health viewpoint since it translates to that many lives can be saved by choosing an optimal vaccination allocation. All outcomes use the same base transmission rate and initial subpopulations in order to obtain a fair comparison. Due to the large number of vaccination strategies tested (3,628,800), it is difficult to observe the optimal ones. However, doing a lot of data scraping from the output files we are able to describe the optimal and worst vaccination strategies. The best 24 vaccination strategies generated similar cumulative number of deaths. This is due to the way vaccines are allocated (mutually exclusive) to the subpopulations and due to rounding in Euler’s method. The order of the first five priority groups for each of these 24 leading strategies is identical: first is the group 0–39 years without comorbidities, then 40–59 years without comorbidities, 40–59 years with comorbidities, 70–79 years with comorbidities, and 60–69 years with comorbidities. The remaining 5 groups are vaccinated after, with slightly different orders depending on the strategy. These best vaccination strategies prioritize the subpopulations with high transmission rates such as the people of working age and in particular the 40–60 age groups. These groups do not necessarily have the higher case fatality rates. Another important result is that the difference in cumulative deaths between the optimal vaccination strategies and the worst performing strategy is approximately 125,000 deaths which is a huge human toll that might occur if the wrong allocation of vaccines is chosen. Our analysis is based only on the outcome metric and does not rely on the transient dynamics of the COVID-19 pandemic before 180 days. Therefore, the effects of the initial subpopulations have been diluted. On the right hand side of Fig 4 is shown a histogram where the distribution of the different vaccination strategies can be seen with regard to the total number of deaths. The distribution is similar to a Gaussian distribution but with a gap probably related to the large number of nonlinearities of the model and the way vaccines are allocated.

### 3.3. Numerical simulations for higher transmission rates scenario

**Figure 5.**
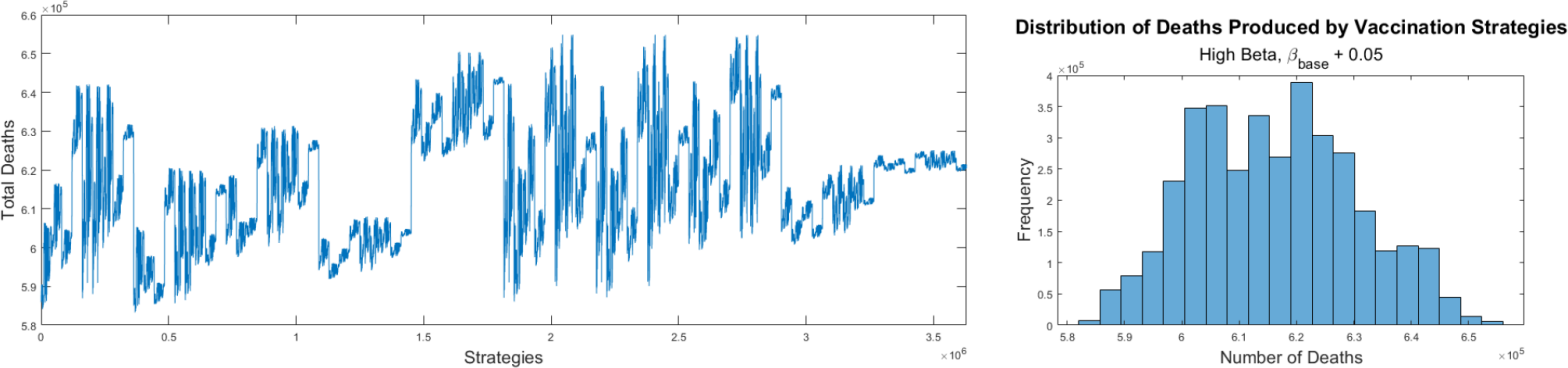
Final cumulative number of deaths for all the different strategies for COVID-19 pandemic vaccination programs using the base transmission rate β≈0.21 (left). Histogram for the different vaccination strategies with regard to the total number of deaths (right).

The second set of numerical simulations that we perform uses larger transmission rates than in the previous section. We increase the base transmission rate β by 0.05 which can be considered a large variation for the transmission rate in the mathematical model (2). Figure 5 shows the final cumulative number of deaths for all the different 3,628,800 potential strategies for COVID-19 pandemic vaccination programs using the base transmission rate β≈0.21. Again, the vaccine strategies produce different outcomes. However, the profile of the graph and therefore the pattern of the optimal vaccine allocations has changed in comparison to the previous results. Due to the large number of vaccination strategies it is difficult to observe the optimal ones, but by data scraping from the output files we can describe the optimal and worst vaccination strategies for this scenario. On the right hand side of Fig 5 the histogram shows the distribution of the different vaccination strategies with regard to the total number of deaths. This distribution is similar to a Gaussian distribution but with one very small spike. In this case the top vaccination strategies each generated a different final cumulative number of deaths. This is due to the fact that at higher transmission rates (faster dynamics), the vaccination strategies become even more important. In this scenario the top vaccination strategies prioritize the subpopulations with higher case fatality rates such as the oldest people and those with comorbidities. For example, the top ten strategies all vaccinate the same four groups first: first 70–79 years with comorbidities, then 80+ years with comorbidities, 40–59 with comorbidities, and finally, 60–69 with comorbidities. These optimal strategies are similar to the actual vaccination strategies implemented by many countries including the USA [16,26,45,52]. Our mathematical approach can be used to analyze different countries or regions by simply modifying the initial subpopulations and the transmission rates related to each country.

In this higher transmission rate scenario the final cumulative number of deaths for all the vaccination strategies is larger than was expected. The differences in the number of deaths in comparison with the previous scenario are approximately 500,000 due the high sensitivity of the epidemiological model (2). Another important result that we would like to emphasize is that the difference between the optimal vaccination strategy and the worst is approximately 72,000 deaths, emphasizing the importance of the vaccination strategy. We performed further simulations (not shown here) with higher transmission rates. We found that the difference in deaths between the best and worst vaccination strategies increased for a β base of 0.26. These simulation results suggest that there could be a minimum for the difference in deaths. Future work can explore this aspect, which is extremely complex due to the large number of nonlinearities in the mathematical model (2) and the procedure to allocate the vaccines. In this study, we focus on the qualitative results and the importance of choosing the optimal vaccine strategy to save human lives.

### 3.4. Numerical simulations for lower transmission rates scenario

**Figure 6.**
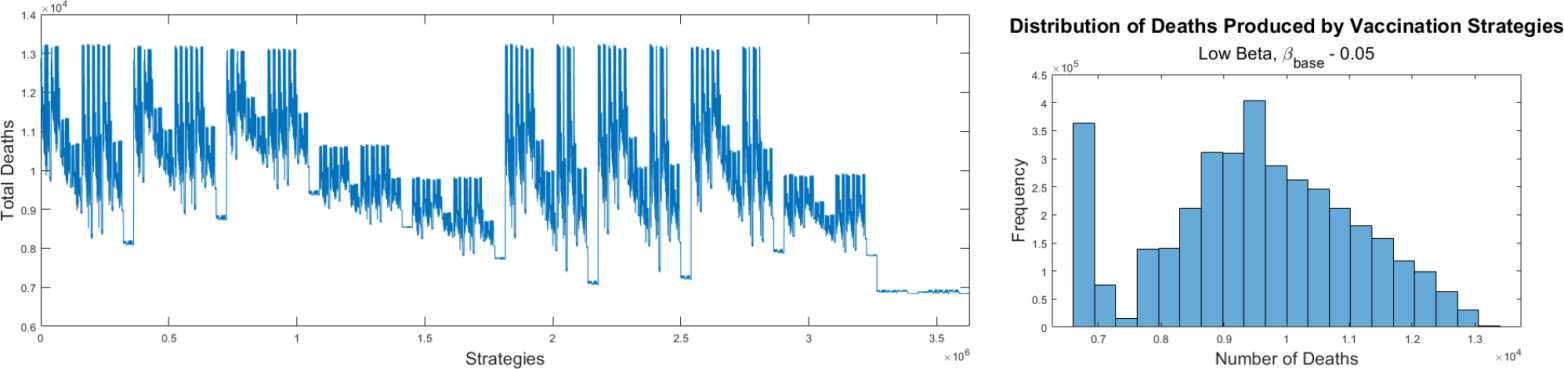
Final cumulative number of deaths for all the different strategies for COVID-19 pandemic vaccination programs using the base transmission rate β≈0.11 (left). Histogram for the different vaccination strategies with regard to the total number of deaths (right).

Finally, the last set of numerical simulations that we perform uses lower transmission. In this simulation we decrease the base transmission rate β by 0.05. Fig. 6 shows the final cumulative number of deaths for all the tested vaccination strategies. As expected, the vaccine strategies generate different final cumulative number of deaths. The profile of the graph and therefore the pattern of the optimal vaccine allocations is different from the previous two tested scenarios. In this case, as in the first scenario, all the top vaccination strategies generate the same final cumulative number of deaths. In this scenario the top vaccination strategies are similar to those in which we used the base transmission rate obtained for the fitting of the model to the real data of deaths. In fact, several of the optimal strategies with the least number of deaths are exactly the same as those found using the fitted base transmission rate. The prioritization is first the subpopulations with higher transmission rates, but also those with higher case fatality rates. The simulation results suggest that in countries where social activity is low the optimal vaccination should shift towards younger people while still taking into account the case fatality rate [153–156].

In this lower transmission rate scenario the final cumulative number of deaths is smaller on average than all the previous scenarios. The difference between the optimal vaccination strategy and the worst is a little above 6,000 deaths. In this regard, we conclude that under very low transmission rates the chosen vaccination strategy is less critical. However, due to the difference in the number of deaths generated in this scenario and the real data it is highly unlikely that the beta transmission was that low in the USA. On the right hand side of Fig 6 the distribution of the different vaccination strategies with regard to the total number of deaths can be seen in the histogram. The distribution is similar to a Gaussian distribution but with a spike at the left hand side probably related to the large number of nonlinearities of the model and the way vaccines are allocated. The spike occurs at a different location than in the case of a higher transmission rate, but similar to the base transmission rate scenario.

### 3.5. Comparison of vaccination strategies for different scenarios

Here we focus on comparing the difference between the best and worst vaccination strategies. The analysis of the details is very complex due to the large number of strategies and the order of the allocation of the vaccines. Therefore, it is difficult to present results in an easy form and with a detailed summary about each vaccination strategy. We present a general summary for the more evident results with the help of previous graphs and the ones presented in this subsection.

**Figure 7.**
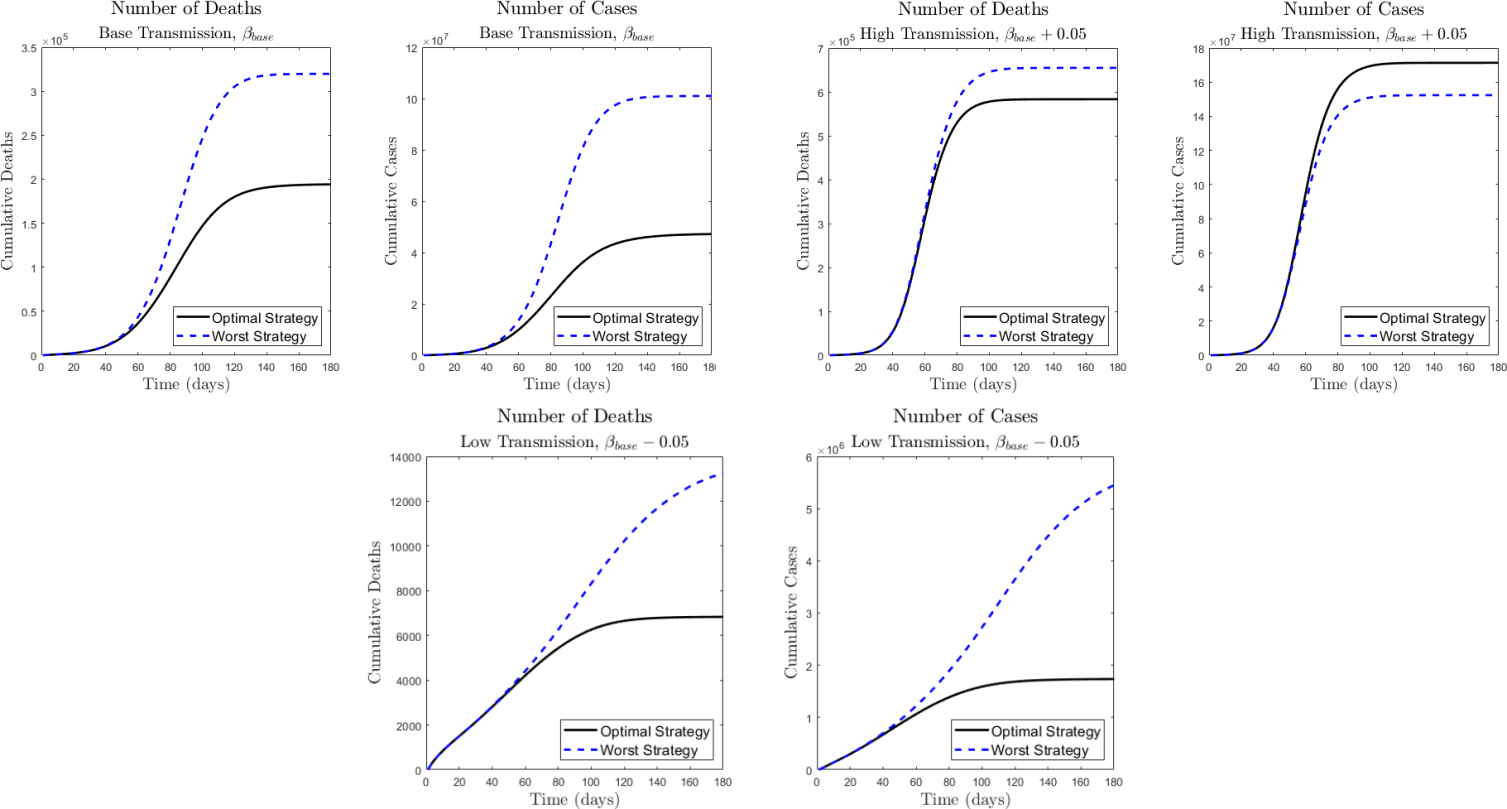
Trajectories for cumulative deaths and infected (total across all subpopulations) corresponding to the best and worst performing vaccine allocation strategies for each of the transmission rate scenarios; base (top-left), high (top-right) and low (bottom).

In Fig. 7 we present the epidemic trajectories for cumulative deaths and infected (total across all subpopulations) as a function of time corresponding to the best and worst performing vaccine allocation strategies for each of the scenarios. A significant difference with regard to deaths and infections just due to the choice of the vaccination strategy can be observed. This emphasizes the importance of studies that investigate the optimal vaccination strategy and that obviously involves the characterization of the particular scenario of the region or country.

**Figure 8.**
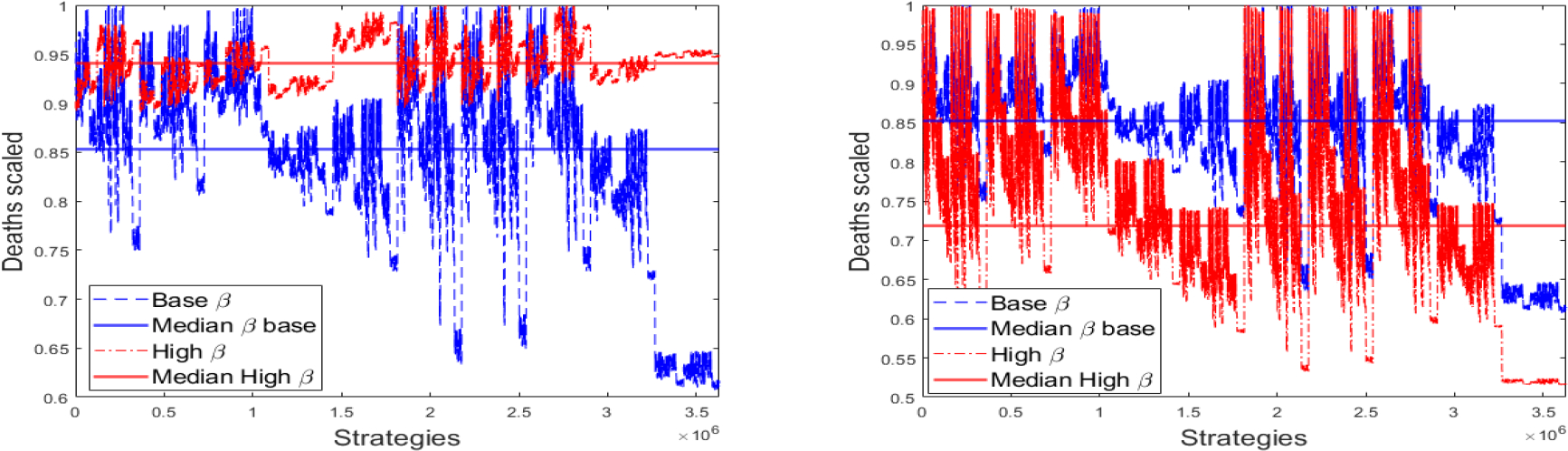
Comparison between the vaccination strategies under different transmission rate scenarios. The total number of deaths is scaled and the medians are shown.

In Fig. 8 we present a comparison between the vaccination strategies under different transmission rate scenarios. On the left hand side we compare the baseline and the high transmission scenarios. We scaled the total number of deaths in order to have a better and fairer comparison. The plot also shows the median of the vaccination strategies based on the total number of deaths. It can be seen that the best vaccination strategies for the baseline scenario are mostly on the right hand side. However, for the high transmission rate scenario those strategies are no longer optimal. Nevertheless, at least they are not the worst. This result partially agrees with what was found in [16] with regard to the CDC vaccination program. On the right hand side we compare the baseline and the low transmission scenarios. It can be observed that the best vaccination strategies for the baseline scenario are similar to those when the transmission rate is low. This result is more promising than the situation faced with the high transmission rate scenario. Table 5 presents a brief summary of these results related to the vaccination strategies under different transmission rate scenarios. For moderate transmission rates the optimal strategy prioritizes high transmission groups with no comorbidities. Notice that we have assumed that people with one or more comorbidities have a reduced transmission rate. For high transmission rate, the dynamics of infections is faster and then the optimal strategy focuses on groups with high CFR in order to avoid deaths. It is important to remark that social contact matrices are different for regions or countries. Therefore, this brief summary can change due to different transmission rates and interconnections between the people from different age groups.

### 3.6. Sensitivity analysis of the initial conditions

**Figure 9.**
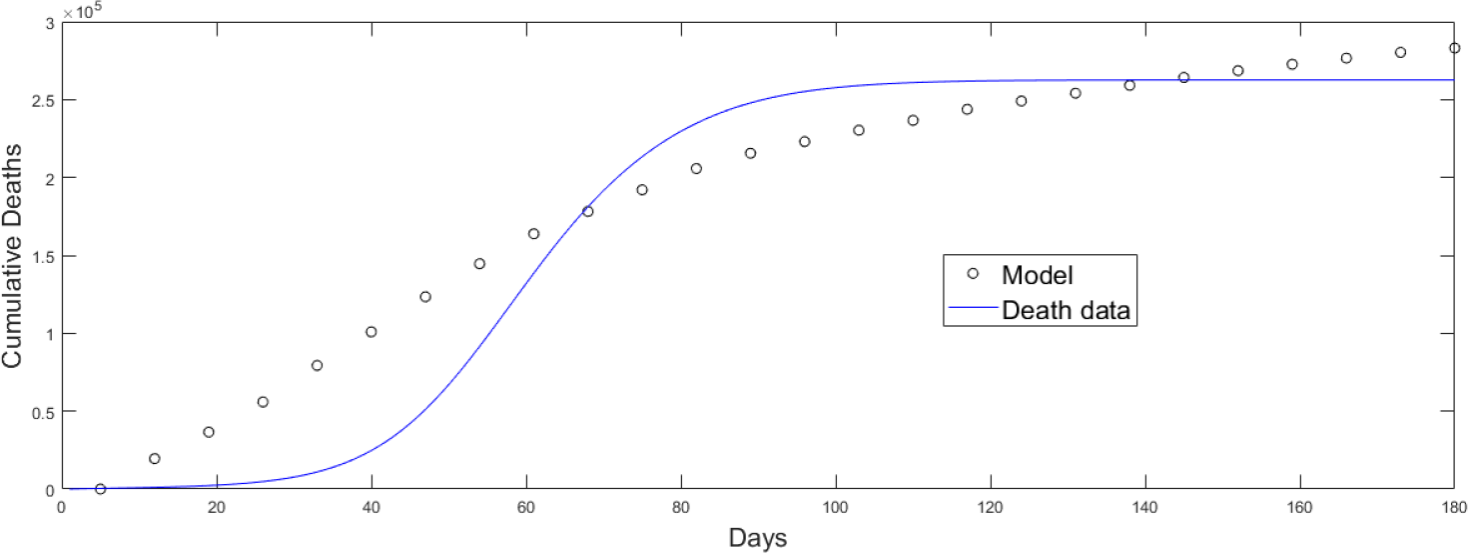
Fitting of the mathematical model (2) to the real data related to deaths but using two times more recovered people for the initial conditions. A new base transmission rate (β≈0.09) is obtained [81].

**Figure 10.**
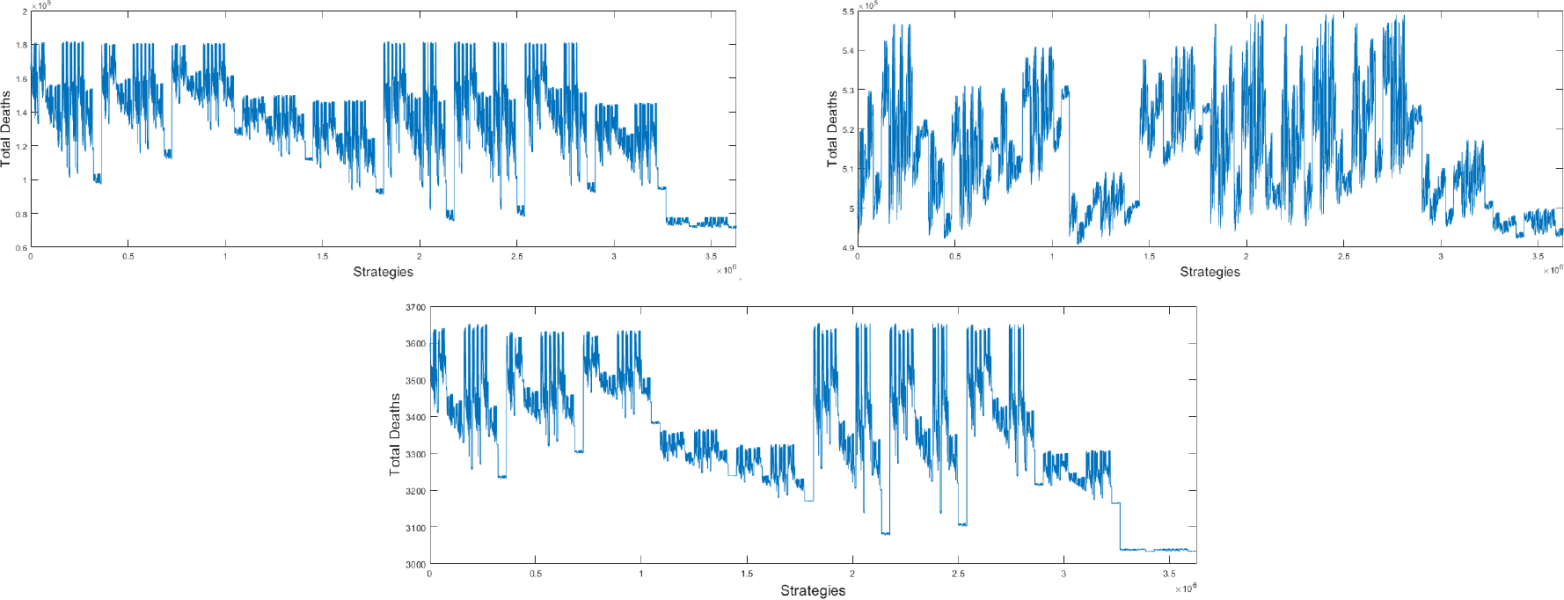
Final cumulative number of deaths for all the different strategies using two times more recovered people for the initial conditions and using previous base transmission rate β≈0.16. The scenarios are baseline (top-left), high (top-right) and low transmission rates.

**Figure 11.**
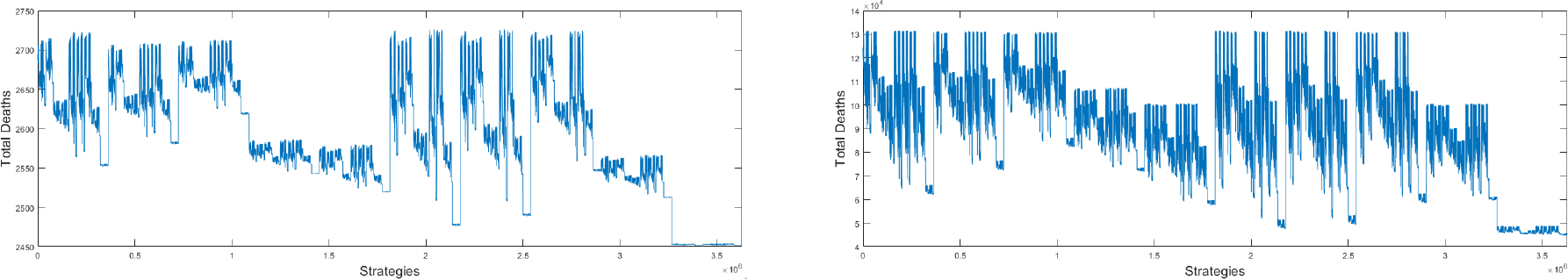
Final cumulative number of deaths for all the different strategies using two times more recovered people for the initial conditions and base transmission rate β≈0.09. The scenarios are baseline (left) and high transmission rates (right).

We performed additional sensitivity analysis by modifying the initial number of recovered and susceptible people. The reason to test this scenario is due to the fact that case count data throughout the pandemic has probably underestimated the actual number of cases [157–159]. Since the study is for short dynamics the initial conditions play a more important role than in classical mathematical studies related to asymptotic behavior. Fig. 9 shows the fit of the mathematical model (2) to the real data related to deaths but using two times more recovered people for the initial conditions. As expected, a new base transmission rate (β≈0.09) is obtained. However, the fit is better than the one obtained with the model that has half of the recovered people. This suggests that more people contracted SARS-CoV-2 than what it was reported. Fig. 10 shows the final cumulative number of deaths for all the different strategies using two times more recovered people for the initial conditions. The patterns of the baseline (top-left), high (top-right) and low transmission rate scenarios are relatively similar to their respective ones that we obtained when using the initial recovered people from the CDC data. However, the pattern for the high transmission rate scenario now is more similar to the baseline transmission rate scenario. We added an additional figure with the new transmission rate that have changed due to the change on the initial conditions. Fig. 11 shows the number of deaths for all the strategies using the high and the base transmission rate scenarios. We didn’t include the low transmission rate case since the number of deaths are very low due to a very slow dynamics of the infections. Again, the patterns are similar to the previous ones. These results provide robustness to the optimal vaccination strategies at the right side of the plots. These vaccination strategies correspond mostly to strategies that vaccinate groups groups with low CFR and high transmissibility.

## 4. Discussion

In this paper, we implemented a mathematical simulation approach to study many different priority-ordered vaccination strategies and find the optimal vaccine allocations in the USA for the early COVID-19 pandemic. We designed a demographic epidemiological mathematical model based on differential equations in order to investigate the efficacy of a variety of vaccination strategies under the limited vaccine supply scenario of the USA. We used the total number of deaths after the first 180 days of vaccine allocation as the metric to measure and compare the efficacy of each of the vaccination strategies. The simulations were performed using 180 days in order to simulate the first 6 months of the vaccination in the USA. The initial conditions were taken from the situation of the USA in December of 2020 when the vaccination roll-out started. Designing the optimal strategy for the vaccination programs is a complex problem due to the large number of variables that affect the outcomes. However, with this work and others similar to it, we aim to provide additional insight into the importance of designing an optimal vaccination strategy. This is crucial since many human lives could be saved. The designed mathematical model takes into account the age, comorbidity status and social contacts of people. We performed a comprehensive simulation study that included more than three million vaccination strategies which vary depending on the vaccination priority of each group. In this study we focused on the scenario corresponding to the early vaccination period in the USA, but the proposed mathematical approach can be extended to other countries by modifying some parameters and the initial conditions for the subpopulations.

For the numerical simulations in each scenario, we assumed time-invariant parameters including the transmission rates of SARS-CoV-2 virus for the multiple groups. This assumption can be justified since the COVID-19 pandemic was already established for more than a year before the period of study and our study focuses on a short time span of six months. Using time-variant parameters can be a future objective that would add further complexity and dimensionality to the study. We used a base transmission rate and from this we computed all the transmission rates for the different subpopulations. These rates are based on a social contact matrix and further reasonable assumptions. For instance, we assumed that vaccine hesitant people are more likely to have more social contacts and people with comorbidities are less likely to have social contacts due to the threat of the Covid disease. The estimation of the base transmission rate is based on a fitting process of the designed mathematical model to the real data of the number of deaths in the USA from December 2020 to June 2021. All the numerical simulations were performed using exactly the same amount of vaccines and allocation rates that were used in the USA [81]. This implies that the availability of vaccines per day was limited. This aspect is important to this study since it differs from a large number of studies related to optimal vaccine allocation where a proportional vaccination rate was assumed.

We evaluated 3,628,800 potential strategies related to the mathematical model (2) under three different SARS-CoV-2 transmission scenarios. The first scenario used the base transmission rate obtained by fitting the model to the real data of deaths in the USA from December 2020 to June 2021. The second and third scenarios used higher and lower base transmission rates, respectively. For the first scenario and the more likely realistic scenario we obtained that in general different outcomes are generated depending on the vaccination strategy. This result is relevant from a public health viewpoint since it shows that, when vaccines are limited, designing an optimal vaccination strategy can save many human lives [160]. In this scenario we found that the best vaccination strategies prioritize the subpopulations with high transmission rates such as the people in working age and people in the 40–60 age group. We also found that the difference between the optimal and worst vaccination strategies was approximately 130,000 deaths. This again shows the importance of designing an optimal vaccination strategy under limited vaccine supply [10, 14, 146, 161]. For the second scenario with higher transmission rates the best vaccination strategies prioritize the subpopulations with higher CFRs such as the oldest people and people with comorbidities. These vaccination strategies were the predominant strategies used by many countries, including the USA [81]. When we doubled the initial number of recovered people the pattern of the optimal strategies shifted closer to vaccinating people with high transmission. Finally, for the last scenario with low transmission rates the best vaccination strategies are similar to the more likely realistic scenario. The prioritization is for the subpopulations with higher transmission rates. All these results suggest that in countries where social activity is low the optimal vaccination should shift towards people with high social contacts but still taking into account the CFR. On the other hand, for countries with higher transmission rates the optimal vaccination strategy should shift towards the people with higher case fatality rates. All these results are under the assumption of a very specific limited vaccine supply which for the best of our knowledge only one study has done but with a different population structure [16]. Overall, our results are in partial agreement with some previous work related to optimal vaccination strategies despite their use of different mathematical approaches [30, 38, 161–163]. It is worthy mentioning that for influenza it has been found that demonstrated that young people is generally robust in the face of uncertainty [138]. However, direct comparisons cannot be made in a strict way since in this study we have used the specific time-varying vaccine supply of the USA and a different population structure. Using a specific time-varying vaccination pace implies short dynamics and theoretical stability analysis cannot be done. Future work can include a more complex and detailed population structure in order to analyze the optimal vaccine ordering strategies. Furthermore, future studies also can include continuous mixing vaccination strategies in order to consider infinitely many strategies.

In our mathematical modeling approach we used a population structure that takes into account age, comorbidities, vaccine hesitancy and social contacts. We obtained a model based on 84 differential equations which requires 84 initial conditions. This requires making some assumptions using some statistics of the USA population. Without assumptions about the transmission rates a better fit can be obtained. However, an issue arises since the parameters would not be identifiable [141, 142, 164]. Another aspect that should be mentioned is that for the fitting process we used a strategy as close as possible to the CDC strategy since our mathematical model has a different population structure than the one used by CDC to implement the vaccination program. Moreover, the CDC used vaccination by phases where in each phase there were multiple groups.

The aim of this study is not to forecast the dynamics of the COVID-19 pandemic or to estimate parameters of the model. The current pandemic has shown that this is a very complex problem due to many factors [17]. One of our aims is to show the importance of optimal vaccination strategies and to show that these optimal strategies change depending on the scenario. Therefore, the optimal vaccination strategy for the USA could be different for other countries. However, we have shown a mathematical approach that can be used for different regions. We have used the number of deaths as a metric to compare vaccination strategies, but other metrics can be used or combined. For instance other studies have considered years of life lost or infected cases.

## 5. Conclusions

Our findings support the idea that optimal vaccine allocation strategies depend on the social contacts or transmissibility of SARS-CoV-2 and that there is not a optimal vaccination strategy for all scenarios [14, 50, 161–163]. The results of this work also provide additional information about the complexity of designing an optimal vaccine ordering strategy for different countries. We found that for low/moderate transmission rates the optimal strategy prioritizes high transmission groups, but for high transmission rates, the optimal strategy focuses on groups with high CFRs. Therefore, each country or region should choose the vaccination strategy depending on their particular scenario related to the transmission rate. For instance, in a country with high amounts of social contacts, it might be better to vaccinate groups with high CFRs first. In some way this was the vaccination strategy used in the USA. Our findings also show that the study of COVID-19 vaccination strategies is of paramount importance to reduce the number of deaths related to the COVID-19 pandemic. The results presented here are qualitative and they are not forecasts regarding the number of deaths over time. It has been proven that accomplishing an accurate forecast of the outcomes of the COVID-19 pandemic is a very challenging problem due to the high variability of the social behavior of people and non-pharmaceutical interventions [165–169].

There are natural limitations in our mathematical approach which occur with any study involving mathematical models. This is due to the attempt to approximate the dynamics of a nonlinear complex real world. The results of using mathematical models are dependent on the assumptions of the model. Therefore, it is important to indicate the limitations of this study to avoid reaching wrong conclusions. Nonetheless, the findings provide useful insights into public health policies and in particular vaccination programs [101, 151, 170–172]. The designed model presented in this work has assumptions. One important assumption is that SARS-CoV-2 transmission rates are time-invariant over the simulation time of six months. We assumed that after more than one year into the COVID-19 pandemic social behavior was approximately time-invariant. The period of study is short in order to avoid having a more complex situation that considers the waning of vaccine efficacy and natural immunity. If the waning of natural immunity against the SARS-CoV-2 is taken into account, then the mathematical model would need modifications. In addition, a more complex mathematical model is necessary if we consider the appearance of new SARS-CoV-2 variants with significantly different transmissibility such the Omicron variant [75, 101, 147, 151, 173, 174]. Another limitation of this work is the uncertainty of symptomatic and asymptomatic cases. We used a conservative approach using CDC scenarios and scientific literature [66, 130, 175]. The transmission rates for each of the subpopulations are also in some way uncertain despite the fact that we performed a fitting to the real data of deaths. There are studies that have shown that when there are many parameters in the model and the available data includes only total deaths and infected cases then there are many sets of parameters that fit the data with the same error [142, 176]. In our model we have a great number of parameters and some assumptions were made in order to fit the data and obtain a unique base transmission rate. For instance, we assumed that the transmission rates are higher for vaccine hesitant people and lower for people with one or more comorbidities. If these assumptions are not valid in reality the results might change. We used a social contact matrix taken from [116, 121], which might not reflect the situation in the USA at the time of the COVID-19 pandemic. However, the fitting process in some way adjusts the social contact rates. There are other further assumptions which are less relevant and common in this type of study.

Finally, the findings obtained in this work encourage governments or public health authorities to characterize the real world situation before designing vaccination programs. We have seen that the optimal vaccine ordering strategy varies depending on factors such as transmissibility of the SARS-CoV-2 virus. Future work should include further analysis of different vaccination programs and to consider the waning of immunity. The results of this study provide additional insight into scientific guidelines for designing optimal vaccination strategies. The findings presented here are based on measuring a particular public health metric, that is, the number of cumulative deaths.

## Figures and Tables

**Table 1.** Initial conditions for the subpopulations [122, 131, 134, 135]

Demographic	Group	Susceptible	Susceptible	Infected	Asymptomatic	Asymptomatic
Age, Comorbidities	Indices	Hesitant	Willing	(Symptomatic)	Hesitant	Willing
	*i*, *k*	*S h_i,k_*(0)	*S w_i,k_*(0)	*I_i,k_*(0)	*Ah_i,k_*(0)	*Aw_i,k_*(0)

0–39 yrs, 0 c	1, 0	52,380,471	66,666,054	32,245	6,081	7,739
40–59 yrs, 0 c	2, 0	6,094,137	29,753,730	8,787	640	3,126
60–69 yrs, 0 c	3, 0	737,463	8,480,829	1,751	60	690
70–79 yrs, 0 c	4, 0	133,912	2,544,321	398	9	162
80+ yrs, 0 c	5, 0	77,314	1,468,974	214	5	87
0–39 yrs, 1+ c	1, 1	19,768,938	25,160,467	12,170	2,295	2,921
40–59 yrs, 1+ c	2, 1	7,388,468	36,073,106	10,653	776	3,790
60–69 yrs, 1+ c	3, 1	2,154,550	24,777,325	5,116	175	2,017
70–79 yrs, 1+ c	4, 1	946,021	17,974,393	2,811	60	1,145
80+ yrs, 1+ c	5, 1	546,189	10,377,592	1,510	32	615
Recovered		12,956,976				

Total Population		326,569,308				

* All initial vaccinated populations are zero at the beginning of the vaccination campaign.

**Table 2.** Parameters’ values used in the simulations.

Parameter	Symbol	Value (Days)

Infectious period	1/*γ*	7 days [145]
Base case fatality ratio	*δ*	0.0019 (calculated) [130]
Base transmissibility	*β*	0.1694 (fitted)
Probability of being asymptomatic	*a*	0.3 [130]
Efficacy of the vaccines	*ϵ_i_*	90% Varied

**Table 3.** Demographic Specific Case Fatality Ratio Factors [117–119].

Demographic Group	CFR Factor	Demographic Group	CFR Factor

0–39 yrs, 0 c	1	0–39 yrs, 1+ c	1.97
40–59 yrs, 0 c	2.53	40–59 yrs, 1+ c	4.98
60–69 yrs, 0 c	7.18	60–69 yrs, 1+ c	14.14
70–79 yrs, 0 c	16.08	70–79 yrs, 1+c	31.68
80+ yrs, 0 c	43.21	80+ yrs, 1+ c	85.12

**Table 4.** Transmission Rates between Demographic Groups [116, 121].

Groups	0–39 yrs, 0c	40–59 yrs, 0c	60–69 yrs, 0c	70–79 yrs, 0c	80+, 0c	0–39 yrs, 1+c	40–59 yrs, 1+c	60–69 yrs, 1+c	70–79 yrs, 1c	80+, 1+c

**0–39 yrs, 0c**	2.1447	1.2562	0.4320	0.2260	0.0746	1.6756	0.9814	0.3375	0.1766	0.0583
**40–59 yrs, 0c**	1.2562	1.9842	0.5501	0.3244	0.1071	0.9814	1.5501	0.4298	0.2535	0.0836
**60–69 yrs, 0c**	0.4320	0.5501	1.0513	0.3613	0.1192	0.3375	0.4298	0.8213	0.2823	0.0932
**70–79 yrs, 0c**	0.2260	0.3244	0.3613	0.5751	0.1898	0.1766	0.2535	0.2823	0.4493	0.1483
**80+, 0c**	0.0746	0.1071	0.1192	0.1898	0.2372	0.0583	0.0836	0.0932	0.1483	0.1853
**0–39 yrs, 1+c**	1.6756	0.9814	0.3375	0.1766	0.0583	1.3090	0.7667	0.2637	0.1380	0.0455
**40–59 yrs, 1+c**	0.9814	1.5501	0.4298	0.2535	0.0836	0.7667	1.2111	0.3358	0.1980	0.0653
**60–69 yrs, 1+c**	0.3375	0.4298	0.8213	0.2823	0.0932	0.2637	0.3358	0.6417	0.2205	0.0728
**70–79 yrs, 1+c**	0.1766	0.2535	0.2823	0.4493	0.1483	0.1380	0.1980	0.2205	0.3510	0.1158
**80+, 1+c**	0.0583	0.0836	0.0932	0.1483	0.1853	0.0455	0.0653	0.0728	0.1158	0.1448

**Table 5.** Summary and characterization of optimal and poor vaccination strategies.

Scenario	Optimal Strategy	Worst Strategy

*β* low	• Initially prioritizes high transmission demographics for the first three groups, then prioritizes demographics with high CFRs. • First vaccinated group: ages 0–39 yrs without comorbidities - the group with the highest number of contacts and the lowest CFR.	• Prioritizes those with low transmission rates first. • Young and working aged individuals (ages 0–39 and 40–59 with and without comorbidities) - those with high transmission rates - are the last four groups to be vaccinated. • First group is 80+ without comorbidities.
*β* base	• Identical to low *β* optimal strategy.	• Initially prioritizes older populations - those with high CFR and low transmission rates. • In general, among groups of the same age those without comorbidities are vaccinated before those with comorbidities.
*β* high	• Prioritizes high CFR groups, but transmission rate of the group is still a factor. • First group is 70–79 with comorbidities, followed by 80+ with comorbidities (the 2 groups with highest CFR and lowest transmissibility).	• First group is 80+ without comorbidities (high CFR, low transmissibility), then 60–69 without comorbidities (mid for both CFR and transmissibility), then 0–39 with comorbidities (relatively high transmissibility and low CFR). • Prioritizes high transmissibility groups significantly more than the optimal strategy.
